# Case study: Methadone maintenance treatment in Hanoi, Vietnam

**DOI:** 10.1186/1477-7517-9-26

**Published:** 2012-07-09

**Authors:** Melissa Jardine, Van Anh Thi Nguyen, Thu Hong Khuat

**Affiliations:** 1 Nossal Institute for Global Health, The University of Melbourne, Melbourne, Australia; 2 Institute for Social Development Studies, Hanoi, Vietnam

## Abstract

Methadone maintenance therapy (MMT) was introduced in the rapidly developing semi-rural district of Tu Liem in Hanoi in December, 2009. Commune police play an integral role in determining which injecting drug users (IDUs) are eligible to commence and continue MMT. This case study highlights the importance of providing training to commune police about MMT to mitigate negative impacts drug law enforcement can have on IDU accessibility to MMT programs.

## Introduction

Methadone maintenance therapy (MMT) was introduced in the rapidly developing semi-rural district of Tu Liem in Hanoi in December, 2009. Located at the District Health Centre, the MMT program complemented a variety of other HIV prevention services being offered including the distribution of clean needles, syringes and condoms, as well as HIV testing and counselling and peer education for people who are at higher risk of HIV transmission, specifically injecting drug users (IDUs). Initially, only 15 IDUs were permitted to commence MMT in 2009, but by March 2012, the MMT program had expanded to include 250 clients. Health centre staff hoped the program would be expanded further.

## Impact of commune police on MMT accessibility

"“The commune police are the most important because they directly monitor, filter and choose which IDUs will be selected for the MMT program” (District Health Centre staff)"

The role of commune police in drug law enforcement conflicts with their legislated role in referring IDUs to MMT. Police in Vietnam have to meet annual quotas of IDUs sent to compulsory detention centres. Commune police reported that these quotas were becoming more difficult to meet following the introduction and expansion of MMT in Tu Liem. Whilst police from both the district and commune level regarded the overall responsibility of the MMT program to be of the Ministry of Health, the commune police are firmly linked to the processes surrounding MMT accessibility for IDUs because it is their role to compile lists of IDUs who are eligible for MMT.

District Health Centre staff reported that commune police were the most crucial to the MMT program because they acted as a ‘filter’ in determining who is not only permitted to commence, but continue, using MMT. The filtering process is carried out by commune police in a number of ways. Initially, commune police distinguish between ‘good’ and ‘bad’ IDUs (the distinction is described further below) and compile a list of those eligible for MMT. The commune police influence selection for MMT by placing ‘good’ IDUs at the top of the list - who are subsequently considered above all others. The commune police present the list to the Commune Steering Committee (comprised of a number of local authorities) for approval (which appears to be a rubber stamp process) prior to being sent to the District level Steering Committee for final selection. Thus, the commune police play a central role in determining which IDUs receive approval to access MMT in Tu Liem.

It is also the role of commune police to monitor MMT clients to ensure they abstain from drug use and abide by clinic regulations. The result of an infraction against MMT regulations usually results in the IDUs being sent to compulsory detention by the commune police.

## ‘Good’ versus ‘bad’ injecting drug users

"“The guidelines [for selection of IDUs for MMT] are unclear, so I have to be flexible when choosing [IDUs] at the commune because I will put the ‘good’ IDUs at the top of the list and the ‘bad’ IDUs at the bottom” (Commune Policeman, Tu Liem District)"

Police interviewed at the commune level in Tu Liem reported categorising IDUs as either ‘good’ or ‘bad’. ‘Good’ IDUs were considered people who, whilst addicted to drugs, were not known to commit crimes such as theft or robbery to support their addiction, nor did they cause arguments or violence amongst their family, or cause social order problems. ‘Bad’ IDUs were people the police knew or suspected of being engaged in some or all of the aforementioned behaviours. In practical terms, the distinction, or discrimination, between these two categories is that commune police effectively ‘reward’ IDUs for their ‘good’ behaviour by enabling them to access MMT, whilst IDUs considered problematic were excluded. When questioned about increasing the overall outcomes of MMT in Tu Liem by prioritising ‘bad’ IDUs for the program, commune police responded by saying ‘bad’ IDUs rarely change their criminal or disruptive behaviour, even after MMT, so investing in their rehabilitation was not worthwhile.

## Social benefits of MMT prioritised over health benefits in Tu Liem

"“Before [MMT], almost every day there were reports of theft cases but now there are almost no cases that IDUs steal or rob from their families, neighbours or in the community” (Commune Policeman, Tu Liem District)"

Of those interviewed for the case study from the health sector, police and IDUs, not one person said that the primary benefit of MMT was HIV prevention. The three most common responses were crime prevention, reduced family violence and improved social order. Interestingly, these were also the three primary benefits reported by the IDUs interviewed. Other reported benefits included, the ability to return to a normal life which included work and not arguing with family about money, improvements in physical health and appearance, specifically changes to the face, particularly to the skin and mouth.

## Challenges

Pressure for commune police to meet quotas for both compulsory detention and MMT presented a conflict for police and the Steering Committees in determining which IDUs are given approval to access MMT. Both commune police and health sector staff reported that the criteria defining who should be given access to MMT were so vague that it gave police wide-ranging discretion as to who was selected. As described above, the concept of ‘good and ‘bad’ IDUs was a discriminatory practice used by commune police to give some IDUs preference over others.

"“I don’t think IDUs should go directly to the clinic…if they go directly to the clinic, we won’t know who can go to the [compulsory detention centre].(Commune policeman, Tu Liem District)"

Currently, there is no option for IDUs to directly access MMT at the health centre – which would remove the need for police involvement in approving eligibility. Changing the legal framework to enable direct access is problematic. In Vietnam, IDUs have to be registered with their local commune police who subsequently monitor their behaviour and try to promote abstinence. In Tu Liem, commune police described MMT as being an ‘alternative measure’ and was referred to as being another method by which police could monitor or manage IDUs, rather than being a form of health treatment – presenting problems for the separation of MMT management from the scrutiny of police.

District Health Centre staff believed that if commune police were educated about the nature of addiction they might be more inclined to overlook minor breaches of the MMT program regulations, such as if MMT clients are caught occasionally using drugs resulting in being sent to compulsory detention by commune police. Furthermore, training for commune police could encourage police to acknowledge that IDUs, even those perceived as ‘bad’, can be rehabilitated and therefore given approval to benefit from MMT.

## Competing interests

The authors declare that they have no competing interests.

## Authors’ contributions

MJ was primary researcher responsible for field research, analysis and writing. THK and VATN responsible for co-ordination and liaison. All authors read and approved the final manuscript.

